# Automatic microarray image segmentation with clustering-based algorithms

**DOI:** 10.1371/journal.pone.0210075

**Published:** 2019-01-22

**Authors:** Guifang Shao, Dongyao Li, Junfa Zhang, Jianbo Yang, Yali Shangguan

**Affiliations:** Department of Automation, Xiamen University, Xiamen, China; Nanjing University of Information Science and Technology, CHINA

## Abstract

Image segmentation, as a key step of microarray image processing, is crucial for obtaining the spot expressions simultaneously. However, state-of-art clustering-based segmentation algorithms are sensitive to noises. To solve this problem and improve the segmentation accuracy, in this article, several improvements are introduced into the fast and simple clustering methods (K-means and Fuzzy C means). Firstly, a contrast enhancement algorithm is implemented in image preprocessing to improve the gridding precision. Secondly, the data-driven means are proposed for cluster center initialization, instead of usual random setting. The third improvement is that the multi features, including intensity features, spatial features, and shape features, are implemented in feature selection to replace the sole pixel intensity feature used in the traditional clustering-based methods to avoid taking noises as spot pixels. Moreover, the principal component analysis is adopted for various feature extraction. Finally, an adaptive adjustment algorithm is proposed based on data mining and learning for further dealing with the missing spots or low contrast spots. Experiments on real and simulation data sets indicate that the proposed improvements made our proposed method obtains higher segmented precision than the traditional K-means and Fuzzy C means clustering methods.

## Introduction

Microarray is a kind of useful biotechnological tool and has been widely applied in the field of life sciences, such as cancer research, pharmacology, disease diagnosis, and environmental engineering [1]. Microarray technology has the advantage of allowing scientists to measure the expression levels of thousands of genes simultaneously. It involves sample preparing, microarray designing, scanning, image processing, and data analyzing [2, 3]. Among them, image processing plays an important role in extracting the gene expressions. Microarray image processing is mainly included by four parts: 1) pre-processing, 2) gridding, 3) segmentation, and 4) intensity extraction [3]. Pre-processing is mainly aimed at reducing the noises and enhancing the image quality. Gridding is implemented to find out a series of horizontal and vertical lines so as to separate one slide image into sub-grids and individual spots areas. The procedure of segmentation divides the spot pixels into foreground, background, or noise. Finally, the intensity extraction is aimed at obtaining the gene expression levels according to the previous operational results. During the four procedures, the segmentation is quite crucial for accurately extracting gene expressions. However, it is a challenging task because the real microarray image usually contains noises, poor contrast and various spot shapes.

(In the past decades, lots of state-of-art tools, such as GenePix [4], Imagene [5], QuantArray [6], ScanAlyze [7], Magic Tool [8], Spot [9], Dapple [10], Spotfinder [11], P-Scan [12], UCSF Spot [13], have been proposed for microarray image processing. They are originally manual and now semi-automatic. In addition, since 1997, microarray image segmentation has attracted increasing attention due to the fact that it is an essential step for extracting gene expression values, and many segmentation algorithms have been proposed for microarray image segmentation. These segmentation methods can be classified into the following seven categories:

1. Shape-based segmentation, including fixed circle or adaptive circle, adaptive shape [14], active contours [15–17] and Snake Fisher model [18]. It can obtain the boundary and region information of spots based on the relativity of target shape;
2. Model-based segmentation, involving Markov Random Filed [19], 3D spot modeling [20] and total variation (TV)-based regularization method [21]. It segments spots by building spot model or modeling the image as a function according to a series of parameter estimation;
3. Region-based segmentation, containing seeded region growing (SRG) algorithm [22] and watershed algorithm [23]. The spots is segmented by splitting image into areas depend on the image topology;
4. Threshold-based segmentation, including global and local threshold [24], soft-thresholding [25]. This method separates foreground and background based on fluorescence intensities.
5. Morphology-based segmentation, involving mathematical morphological method [26–27]. It segments spots using morphological operations with various structuring element.
6. Supervised learning-based segmentation, containing neural networks [28] and support vector machines [29]. These methods segment the spots depended on prepared training dataset.
7. Unsupervised learning-based segmentation, including K-means clustering [30–31], improved K-means clustering [32–34], Fuzzy C means clustering [35–39], and other clustering methods, such as expectation-maximization [40], model-base clustering [41], combined clustering [3,42], genetic algorithm combined clustering[43]. This kind of methods segment spots based on the natural relationship between intensities.

However, among these methods, the clustering-based methods are prone to be affected by noises. Meanwhile, real microarray images usually contain poor quality problem such as low contrast, noises, artifacts, shape-varied spots, and so on. All these deficiencies make the clustering-based segmentation become a challenging task. Therefore, in this article, an improved clustering-based scheme is proposed to improve the segmentation efficiency. Firstly, an improvement of automatic contrast enhancement and noise reduction is introduced into the image preprocessing. Subsequently, an initial clustering center design scheme is proposed for improving the clustering performance instead of random points selecting. Because the single feature of intensity cannot reflect all characters of spots, the factors of spatial, intensity and shape features are considered. In addition, the principal component analysis (PCA) is implemented for feature selection. Finally, an adaptive adjustment strategy is adopted for improving the segmentation accuracy. This article is organized as follows. Section 2 introduces the improved clustering-based method. Section 3 presents the comparison experiments on different data sets. The main conclusions are described in Section 4.

## The improved clustering-based method

**Fig 1:**
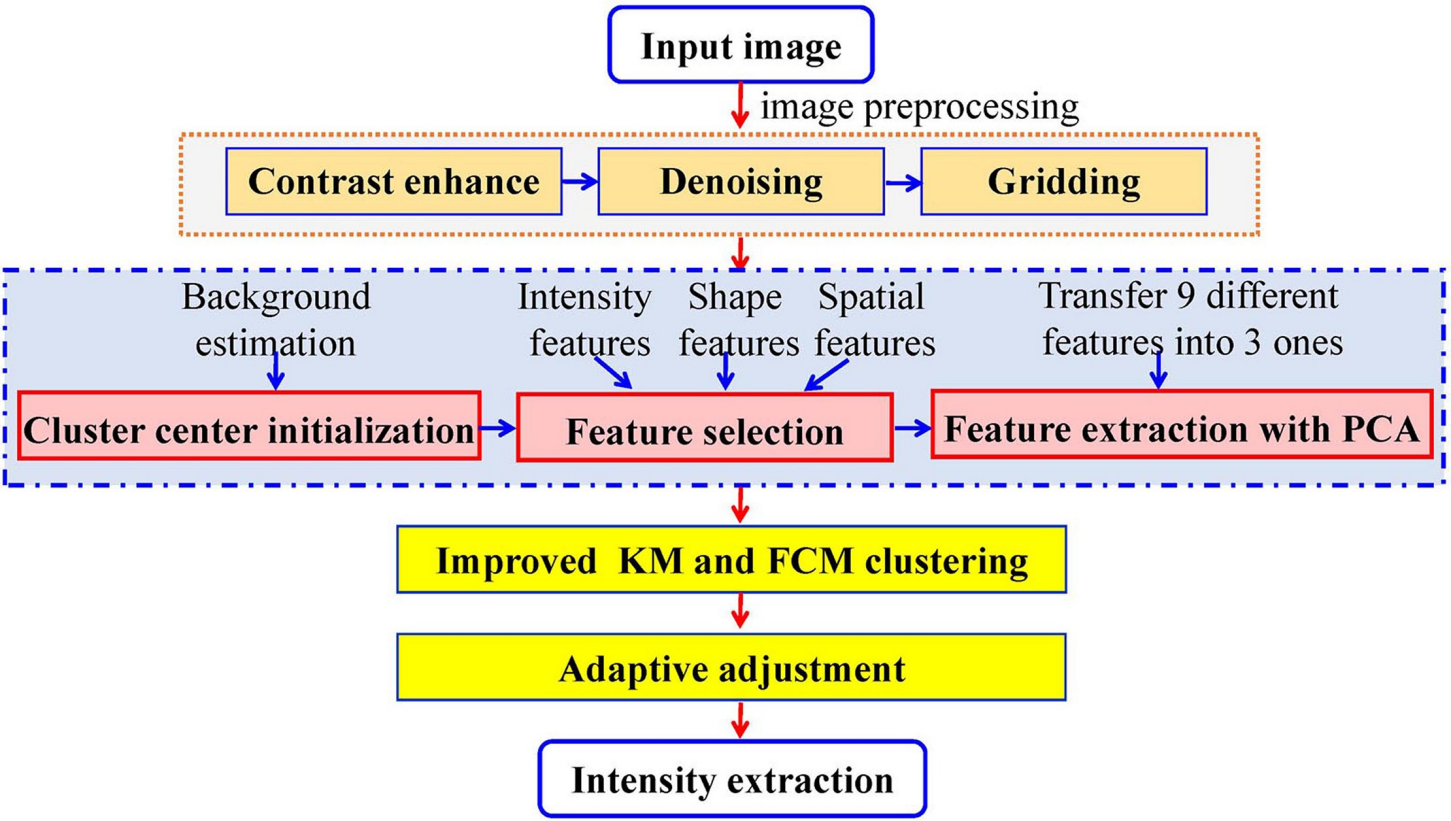
Flowchart of improved clustering-based image segmentation method.

As aforementioned, a great number of clustering methods have been proposed for cDNA microarray image segmentation. However, only a portion of them perform well on simulation images. In addition, single intensity feature is adopted for clustering in almost all the algorithms. To improve the segmentation accuracy and take full advantage of traditional clustering method, we improved the KM and FCM clustering algorithms. The proposed method (online code is in S1 Algorithm) includes six parts: 1) image preprocessing, 2) cluster center initialization, 3) feature selection, 4) feature extraction with PCA, 5) improved KM and FCM clustering, 6) adaptive adjustment, as shown in Fig 1.

### Image preprocessing

To enhance the image quality, the contrast enhancement and gridding are conducted during the image preprocessing step. Our previous method has been adopted for contrast enhancement [3], and it can be executed according to the following steps:

1. Computing image contrast by using the four-order moment *C* = *Sd*/[*Fom*/*Mse*^2^]^1/4^, in which $Sd=\sqrt{\frac{1}{N}\left(\sum {\left(p-\bar{p}\right)}^{2}\right)}$ denotes the standard deviation, $Mse=\frac{1}{N}\sum {\left(p-\bar{p}\right)}^{2}$ represents the mean square error, $Fom=\frac{1}{N}\sum {\left(p-\bar{p}\right)}^{4}$ is the four-order moment and $\bar{p}=\frac{1}{N}\sum p$ is the mean value of the image, *N* is the number of image pixels. For example, the cDNA microarray images drawn from GEO data set are generally low contrast, and their *C* values are all under 1000.
2. Estimating background gray value with the proposed method, that is $k=\frac{1}{m}\sum_{i=0}^{m}\underset{i}{\min}\begin{array}{c} \left\{\underset{j}{\max}A_{j}\right\} \end{array}(j\in [1,12])$, in which *m* is the repeated experiments times, *A*_*j*_ is a 10*10 window randomly selected from four borders of the image.
3. Enhancing the image contrast by using the following operation $g(x,y)=\{\begin{array}{cc} f(x,y)\times (10000/C) & f(x,y)\leq k\\ f(x,y) & otherwise \end{array}$.

After contrasting the enhancement operation, a median filter is applied for noise reduction.

In addition, the maximum between-class variance based gridding is conducted according to the following steps [3]:

1. Computing the horizontal or vertical projection signal;
2. Filtering the projection signal by morphological reconstruction;
3. Finding the optimal threshold by maximum between-class variation operation;
4. Obtaining the horizontal or vertical grid lines according to the thresholding;
5. Revising the gridding result based on the heuristic rules;
6. Acquiring the final coordinate vector *H* and *V* for all grid lines, meanwhile obtaining the horizontal grid line number *h* and the vertical grid line number *v*.

**Fig 2:**
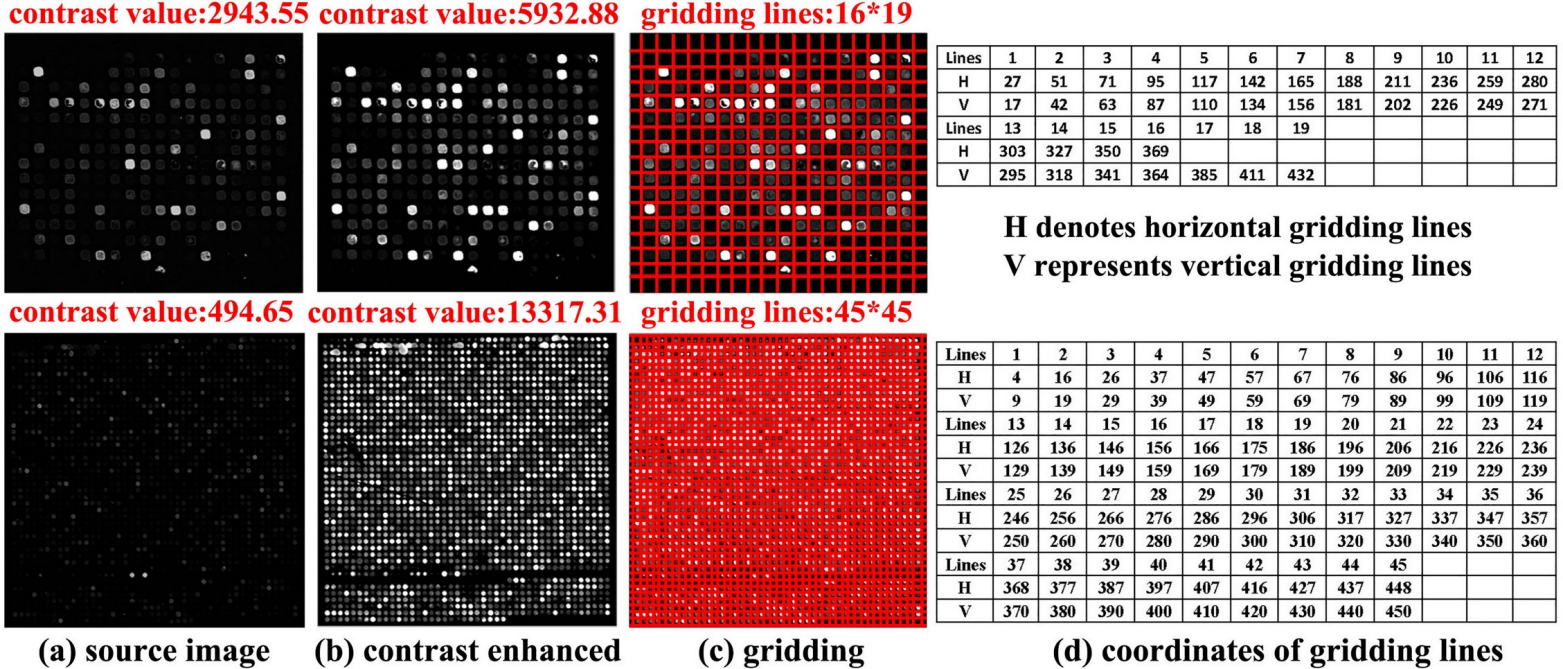
Examples of image preprocessing with (a) source image, (b) contrast enhanced image, (c) gridding image, and (d) coordinates of gridding lines.

Examples of image preprocessing are shown in Fig 2. It can be seen from Fig 2 that the image contrast is greatly enhanced and the correct gridding results are obtained.

### Cluster center initialization

The class number and the initial cluster center are two vital factors for obtaining good clustering performance. Here we set the number of clustering classes to 2. For the initial cluster center of one group, we put forward to adopt the background gray value *k* estimated in the image preprocessing step instead of random initialization. As for another group, the gray mean of a 3*3 window in each spot center is selected as the initial cluster center.

### Feature selection

Generally, the pixel intensity features are adopted by various clustering algorithms and the classification is realized according to the Euclidian distance between the pixel and cluster center. If the intensity of a pixel is high, it will have a great probability to belong to the spot and vice versa. However, there exists the missing classification situation since the noises may contain higher intensities. Therefore, here we implemented other features, including intensity features, spatial features and shape feature, as shown in Table 1. To be specific, the intensity features are composed of the pixel intensity, the mean intensity, the intensity standard deviation and the entropy [29]. The spatial features include the coordinates of each pixel, the Euclidean distance and the city block distance between the pixel and the clustering center. In addition, to describe the similarity between the pixels centered area and the theoretical spot, the shape feature which computes the correlation coefficient is adopted. In the shape feature, the neighborhood size of pixel and the size of Gaussian template is $d=\frac{1}{2}\left(\frac{1}{v}\sum_{i=1}^{v}\left(V_{i+1}-V_{i}\right)+\frac{1}{h}\sum_{j=1}^{h}\left(H_{j+1}-H_{j}\right)\right)$, obtained from gridding step, *H* and *V* denotes the coordinates of horizontal and vertical gridding lines, respectively.

**Table 1:** Features used in our approach.

Type	Form		Description	
**Shape feature**	*r*		$r=\frac{\sum {}_{i}\sum {}_{j}(I(i,j)-\bar{I})(T(i,j)-\bar{T})}{\sqrt{(\sum {}_{i}\sum {}_{j}{\left(I(i,j)-\bar{I}\right)}^{2})(\sum {}_{i}\sum {}_{j}{\left(T(i,j)-\bar{T}\right)}^{2})}}$, *T*(*i*,*j*) is a Gaussian template with variable size of *d*×*d*, *d* represents the estimated spot diameter	
**Spatial features**	*i*		Row of the pixel	
	*j*		Column of the pixel	
	*D*_*M*_ = |*i*−*i*_*c*_|+|*j*−*j*_*c*_|		City block distance of each pixel to clustering center, *i*_*c*_, *j*_*c*_ are row and column of clustering center	
	$D_{Eud}=\sqrt{{\left(i-i_{c}\right)}^{2}+{\left(j-j_{c}\right)}^{2}}$		Euclidean distance of each pixel to clustering center	
**Intensity features**	*I*(*i*,*j*)		Intensity of pixel (*i*,*j*)	
	$\bar{I}(i,j)=(1/9)\sum_{i=1}^{3}\sum_{j=1}^{3}I(i,j)$		Mean intensity value of the 3*3 pixel centered window	
	$\delta =\sqrt{\frac{1}{9}\sum_{i=1}^{3}\sum_{j=1}^{3}{\left(I(i,j)-\bar{I}\right)}^{2}}$		Intensity standard deviation of the 3*3 pixel centered window	
	$E=\sum_{m=0}^{I_{\max}}p_{m}\log_{2}(1/p_{m})$		Entropy of a 3*3 pixel centered window, *p*_*m*_ is histogram counts, *I*_max_ is maximum gray value of the image	

According to the definition above, a feature vector $F={\left[r,i,j,D_{M},D_{Eud},I(i,j),\bar{I}(i,j),\delta ,E\right]}^{T}$ of each pixel can be obtained. However, how to use these different features is quite important and no literatures discussed the multi-features fusion scheme for microarray image segmentation to date. Therefore, we deal with these features by using PCA which transfers a set of correlation variables into another irrelevance one by linear transformation. According to the covariance eigenvalue output from PCA operation, the top three transformed principal components are selected. Finally, the Euclidean distance is adopted for evaluating the relationship between the clustering center and each pixel.

### Improved KM and FCM clustering

As KM clustering is a kind of general algorithm, therefore, we mainly introduce the operations of FCM clustering here. There are four main steps for conducting FCM clustering.

Step1: Initializing the membership grade function according to $\sum_{i=1}^{c}u_{ij}=1,\forall j=1,\ldots ,N$. *u*_*ij*_ represents the membership degree of pixel *j* to cluster *i*.

Step 2: Updating the membership values for each pixel

$$u_{ij}=1/\left(\sum_{k=1}^{c}{\left(d_{ij}/d_{kj}\right)}^{2/(m-1)}\right).$$ (1)

Where *d*_*ij*_ = ‖*x*_*j*_−*c*_*i*_‖ and *d*_*kj*_ = ‖*x*_*j*_−*c*_*k*_‖ denote the Euclidean distance between the feature vectors of pixel *j* to cluster *i* and *k*.

Step 3: Updating the cluster centroid by

$$c_{i}=\left(\sum_{j=1}^{N}u_{ij}^{m}x_{j}\right)/\left(\sum_{j=1}^{N}u_{ij}^{m}\right),$$ (2)

*m* is the fuzziness parameter.

Step 4: Computing the cost function by the following equation

$$J(U,c_{1},\ldots ,c_{c})=\sum_{i=1}^{c}J_{i}=\sum_{i=1}^{c}\sum_{j}^{N}u_{ij}^{m}d_{ij}^{2}.$$ (3)

Step 5: Repeating step 2–4 until the cost function is minimized.

The detail Pseudo of the proposed FCM algorithm can be seen as following.

**Algorithm 1**

The pseudo code of improved FCM algorithm

**Input:** c: number of clusters.                       Max: maximum number of iterations.

       X: data matrix with selected features.       tol: termination threshold.

       U: membership grade function.                     m: the fuzzy coefficient.

**Output:** Predicted labels of data and cluster centers.

Initializing the cluster centers;

**for** *j*←1 **to** N **do**

     Normalizing U;

**end**

*c*_*old*_←c;

**for** *k*←1 **to** Max **do**

     **for** *i*←1 **to** c **do**

          **for** *j*←1 **to** *N* **do**

               update membership grade function *U* by Eq (1);

          **end**

     **end**

     **for** *i*←1 **to** c **do**

               update the cluster centers *c*_*new*_ based on Eq (2);

**end**

compute *δ* ←max(|*c*_*new*_−*c*_*old*_|);

*c*_*old*_←*c*_*new*_;

**if** *δ*<*tol*

               **quit**;

**end**

**end**

predicted labels ←*U*;

**return;**

### Adaptive adjustment

**Fig 3:**
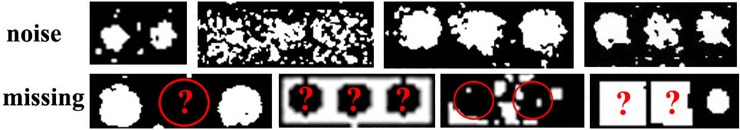
Deficiency examples of different segmentation results.

After clustering by using the method of IKM and IFCM, there may exists some situations as shown in Fig 3. On one hand, the segmented spots may be surrounded by noises, for example, the top line shown in Fig 3. On the other hand, the missing spots appeared with different situations, as the bottom line depicted in Fig 3.

To avoid the deficiencies described above, an adaptive adjustment is crucial step. Therefore, we put forward a method of noise removal and missing spot estimation based on the spot size. Firstly, an approximate spot size is estimated according to the microarray image data. To realize this, we utilize the boundary lines information computed in the gridding step [3]. Let $BH^{0}=\left[H_{1}^{0},\cdots ,H_{bh}^{0}\right]$ and $BV^{0}=\left[V_{1}^{0},\cdots ,V_{bv}^{0}\right]$ represent the boundary grid lines coordinates on horizontal and vertical, respectively. Then we can estimate one spot size by $s_{i}=\frac{1}{2}\left[\left(H_{2i}^{0}-H_{2i-1}^{0}\right)+\left(V_{2i}^{0}-V_{2i-1}^{0}\right)\right]$. Therefore, the rough spot size for one microarray sub-grid image can be obtained by $s=\frac{2}{bh}\sum_{i=1}^{bh/2}\left(H_{2i}^{0}-H_{2i-1}^{0}\right)+\frac{2}{bv}\sum_{j=1}^{bv/2}\left(V_{2j}^{0}-V_{2j-1}^{0}\right)$.

The adaptive adjustment algorithm can be executed according to the following steps:

*Step 1*. Estimate the rough spot diameter *s* by using the results drawn from the gridding step.
*Step 2*. Draw a circle in diameter *s* within each spot area.
*Step 3*. Consider the recognized foreground pixels (the white pixel) outside circle as noises and remove them.
*Step 4*. Count the total pixel number *n*_*s*_ and the recognized foreground pixel number *n*_*f*_ during the rectangular spot area.
*Step 5*. Count the number of recognized foreground pixels *n*_*c*_ inside the circle area.
*Step 6*. A spot is considered as a missing spot when *n*_*c*_<0.5×(3.14×*s*) or (*n*_*c*_≥3.14×*s*) & (*n*_*f*_>0.9×*n*_*s*_). The former indicates that the recognized spot pixels are less than half of the circle area pixel number. The latter one describes the situation that the recognized spot pixels are more than the pixel number in a circle area and the recognized foreground pixel number is more than 90% of pixel number in a rectangular area (see Fig 3). All these illustrate that the segmented results are false.

### Intensity extraction

When the spots are segmented into foreground and background parts by our proposed clustering method, the spot expression value can be obtained according to *E* = log_2_(*I*_*cy*3_/*I*_*cy*5_). In which *I*_*cy*3_ = *R*_*fore*_−*R*_*back*_,*I*_*cy*5_ = *G*_*fore*_−*G*_*back*_ indicates the background corrected spot intensities of Red and Green channels, and *R*_*fore*_,*G*_*fore*_ represent the mean intensities of the foreground pixels, yet *R*_*back*_,*G*_*back*_ denote the mean intensities of the background pixels.

## Image quality assessment

To estimate the performance of our proposed methods, two means are adopted. One is conducting experiments on both the simulation and real images. The other is introducing various quantitative measurements.

### Testing data sets

On the simulation and real images, six real data sets and one simulation data set are adopted, as shown in Table 2. In addition, we used a microarray simulation model (see http://www.gnu.org/copyleft/gpl.html [44]) to generate various quality images.

**Table 2:** Description of six real microarray data sets.

Data set	Full name	No. of blocks	Spot layout in each block	Spot resolution	Image resolution
**SMD**	Stanford Microarray Database	48	14*18 or 44*44	18*18 or 8*8	460*451
**GEO**	Gene Expression Omnibus	48	13*14	12*12	451*391
**BCM**	Bachelor College of Medicine	48	22*22	25*25	943*949
**DeRisi**	Joe DeRisi Individual	4	40*40	8*8	463*455
**SIB**	Institute for Experiment Cancer Research	4	5*7 or 7*7	18*18	366*350
**UCSF**	University of California, San Francisco	36	14*15	8*8	218*209

First, the ScanAlyze toolbox is used to generate real microarray image, and some parameters (gray mean of spot and background intensity) are obtained. Then the gray mean of each spot is taken as the input of simulation model. Meanwhile, the cDNA microarray data can be generated through simulating the hybridization behavior of probe on slide surface and modifying the general options. To control the quality of the simulated image, some model parameters such as different noise model selecting, slide parameter setting, hybridization parameter controlling, and virtual scanner parameter setting, are tuned. Based on the steps above, three types (good, normal and poor) of microarray images are generated.

### Quantitative measurement

To comparative analyze the segmentation results, some quantitative measurements are conducted on the expression level of spots. Firstly, the log differential expression ratio *M* = log_2_(*I*_*cy*3_/*I*_*cy*5_) and the log intensity $A=\frac{1}{2}\log_{2}\left(I_{cy3}\times I_{cy5}\right)$ are introduced. In addition, a quality index [45] *q*_*index*_ = (*q*_*sig*−*noise*_⋅*q_*bkg*1_⋅*q_*bkg*2_)^1/3^ is used and *q*_*sig*−*noise*_ = *F*_*mean*_⋅/(*F*_*mean*_+*B*_*mean*_) represents the signal to noise ratio, the level of the local background is expressed as $q_{bkg2}=\frac{1}{\max\left[\frac{bgk_{0}}{bgk_{0}+B_{mean}}\right]}*\frac{1}{\frac{bgk_{0}}{bgk_{0}+B_{mean}}}$, the local background variability is defined as $q_{bkg1}=\frac{1}{\max\left[\frac{BSD}{B_{mean}}\right]}*\frac{1}{\frac{BSD}{B_{mean}}}$. In which *F*_*mean*_ denotes the mean value of the spot, *B*_*mean*_ is the mean value of the local background, *BSD* indicates the standard deviation of the local background, and *bgk*_0_ represents the global average of the background. In addition, the number of pixels clustered as foreground and background for each spot is represented by *N*_*fore*_,*N*_*back*_, respectively. Furthermore, mean of the expression value *MI*_*cy*3_,*MI*_*cy*5_ for each sub-grid are also introduced.

For the simulation data, owing to its corresponding annotate image, hence we can compute the pixel to pixel accuracy *acc* = (*TP*+*TN*)/(*TP*+*TN*+*FP*+*FN*), the sensitivity *se* = *TP*/(*TP*+*FN*) and the specificity *sp* = *TP*/(*TP*+*FP*). In which, *TP* denotes the correct number of pixels segmented as spot, *TN* represents the correct number of pixels segmented as background, yet *FP* and *FN* indicate the false number that spot pixels are recognized as background and background pixels are considered as spot, respectively.

## Results and discussion

The proposed clustering based algorithms (IKM and IFCM) are compared with traditional KM, FCM methods and moving K-means (MKM) [3] on six different data sets and one simulation data set.

### Image segmentation

**Fig 4:**
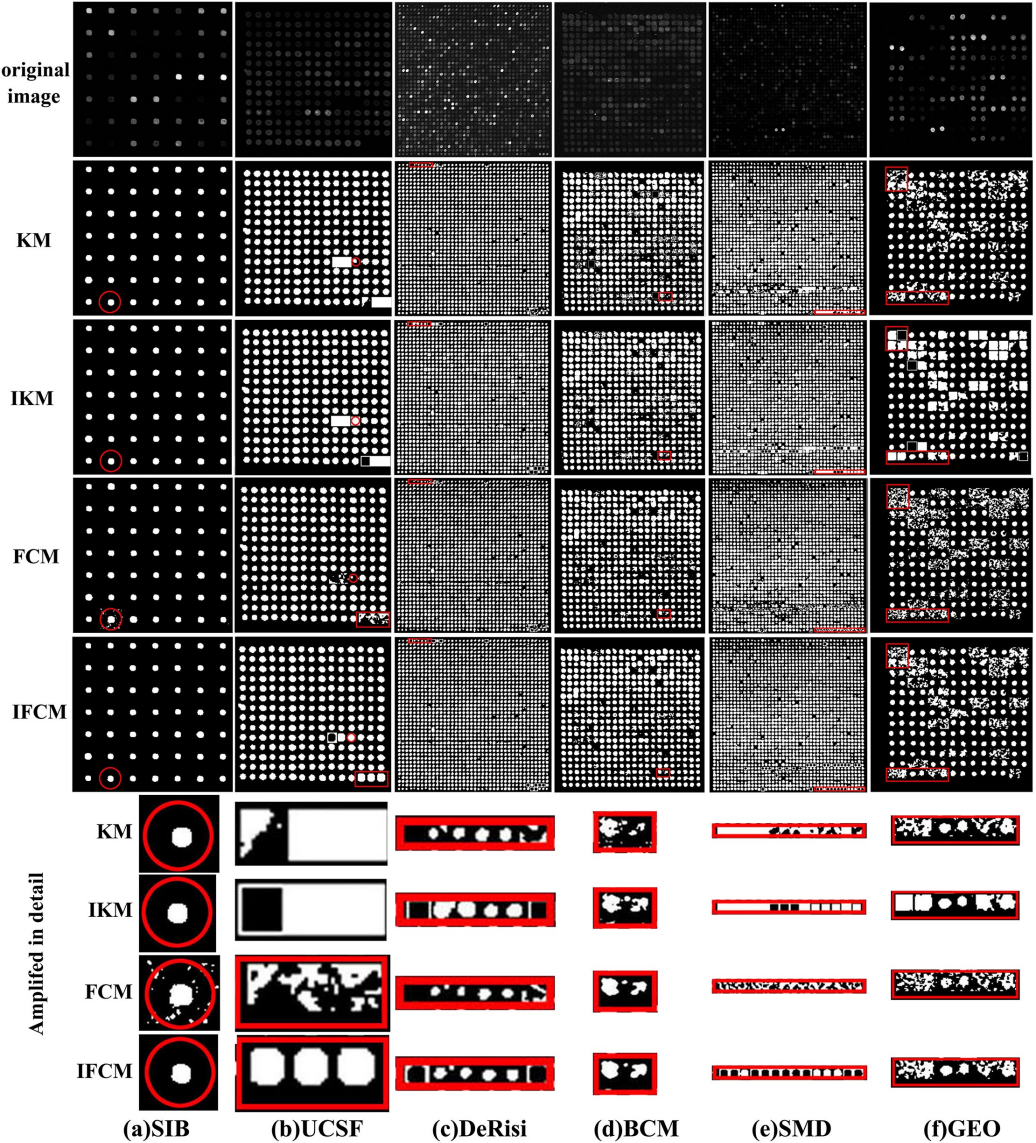
Segmentation results of four methods on microarray images drawn from six data sets.

Performance of four methods is compared on 188 real sub-grids drawn from 6 data sets, and the randomly selected original images and their corresponding segmented results are shown in Fig 4. It can be seen that a majority of spots are separated from background by these four methods. Segmentation results on GEO and SMD data sets are poor due to the low contrast of original images. In contrast, segmentation results on SIB data set are better owing to their high contrast of original images and FCM performs worst due to its sensitiveness to noises. It can be concluded that the performance of these methods is dependent on image quality. Moreover, IKM and IFCM algorithms can extract more low contrast spots than KM and FCM methods. Especially, IKM and IFCM methods can recognize missing spots by our proposed adaptive adjustment method.

**Fig 5:**
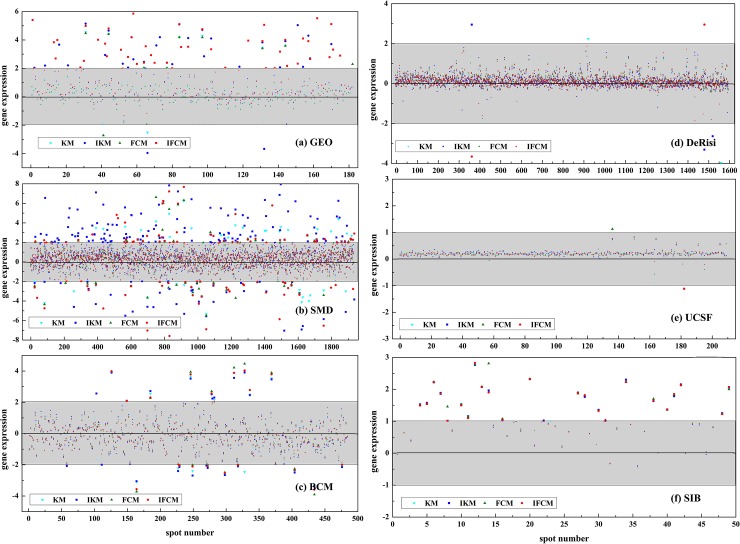
Gene expression for images in Fig 4 with four different methods.

To further compare the performance of four methods, the corresponding gene expression values for images in Fig 4 are presented in Fig 5. Generally, gene expression values between -2 to +2 are considered as normal data, yet those less than -2 or higher than +2 may represent noises or special genes. Therefore, we use a bigger size symbol to describe those special gene expressions as shown in Fig 5.

From Fig 5A–5C, it reveals that gene expression values varies greatly on GEO, SMD and BCM data sets owing to the low contrast of original images. At the same time, there is more gene expressions exceeding +2 or lowering -2 and most of them can be correctly obtained by IKM and IFCM methods, indicating that the improved methods perform better on poor quality image than KM and FCM algorithms. For Fig 5D–5F, a majority of gene expressions are between +2 to -2 due to the high quality of original images. In other words, almost all the spots are correctly segmented by these four methods.

In addition, the number of gene expression beyond +2 and -2 are counted for different methods on 6 data sets, as shown in Table 3. It can be seen that IKM and IFCM algorithms obtain more special gene expressions than KM and FCM ones. Especially for SMD and GEO data sets, partial spots cannot be extracted by KM and FCM methods due to their poor quality. Therefore, there is a lot of special gene expressions obtained by IKM and IFCM.

**Table 3:** Special gene expression comparison of four methods on 6 data sets.

Data set	No. of gene expression>2				No. of gene expression<-2			
	KM	IKM	FCM	IFCM	KM	IKM	FCM	IFCM
**GEO**	8	31	9	50	1	3	1	0
**SMD**	51	129	30	55	29	45	28	42
**BCM**	8	12	7	11	12	12	5	8
**DeRisi**	1	1	0	1	1	4	0	1
**UCSF**	0	0	0	1	1	1	0	0
**SIB**	7	7	8	7	0	0	0	0

### Quantitative analysis

**Fig 6:**
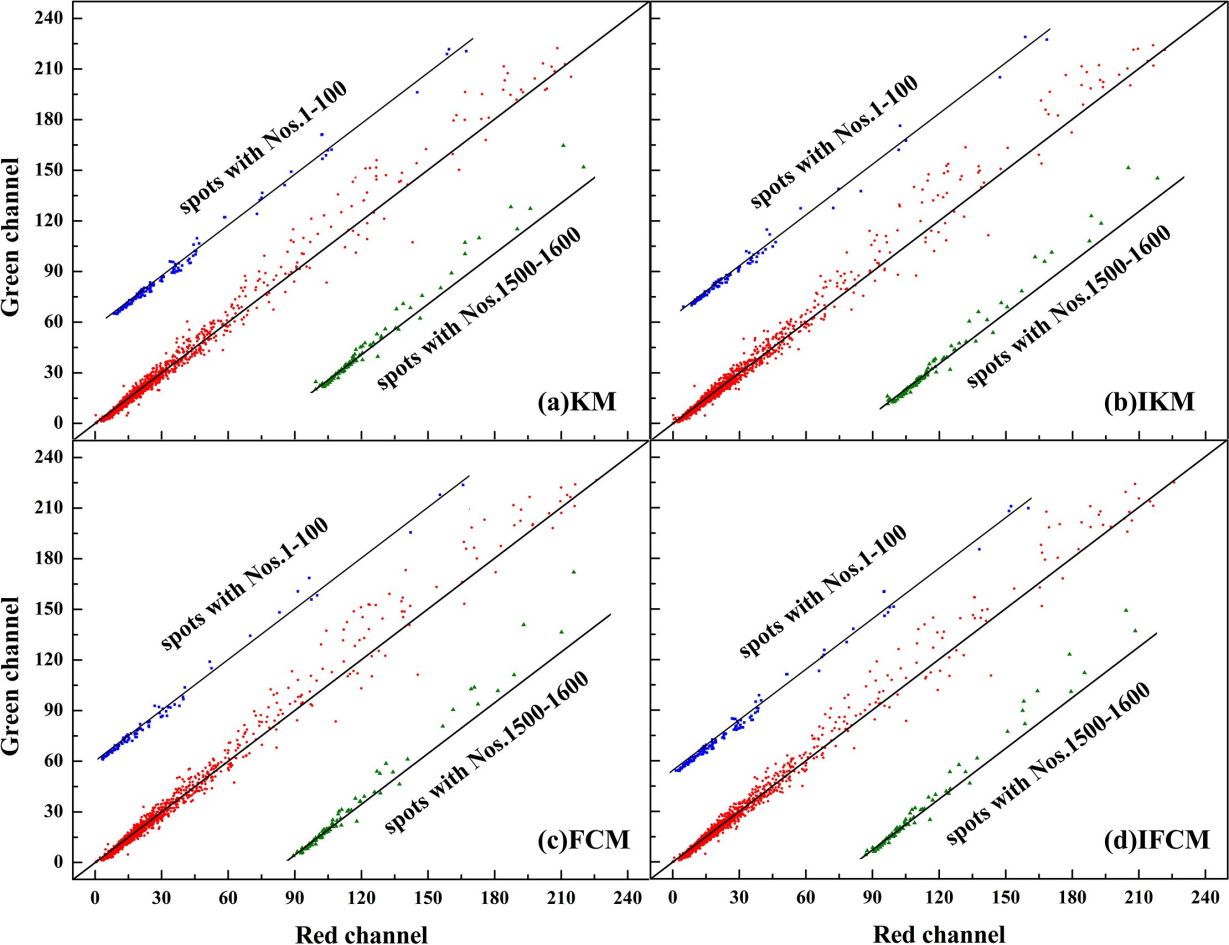
Scatter plot of two channel intensities for four methods on image drawn from DeRisi dataset.

**Fig 7:**
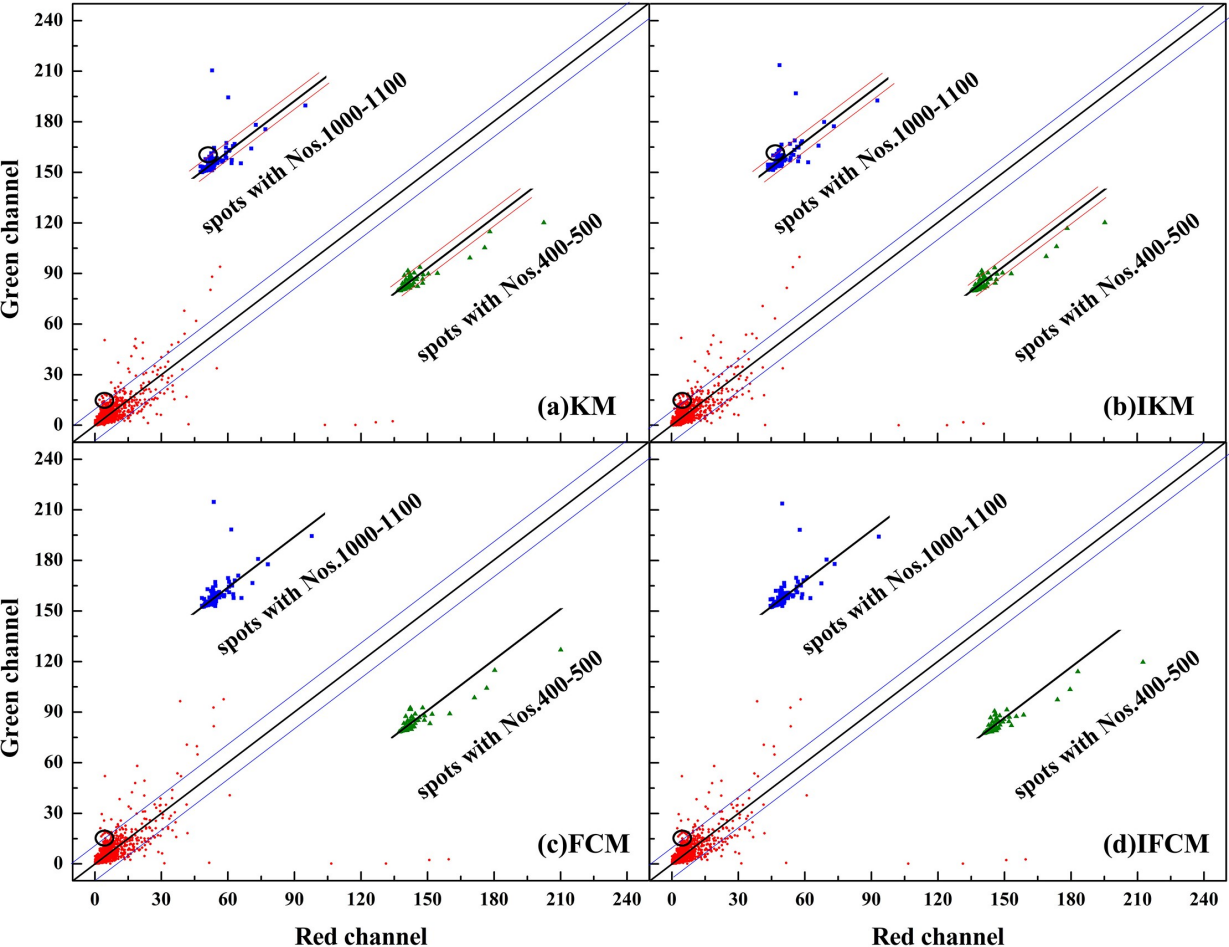
Scatter plot of two channel intensities for four methods on image drawn from SMD dataset.

**Fig 8:**
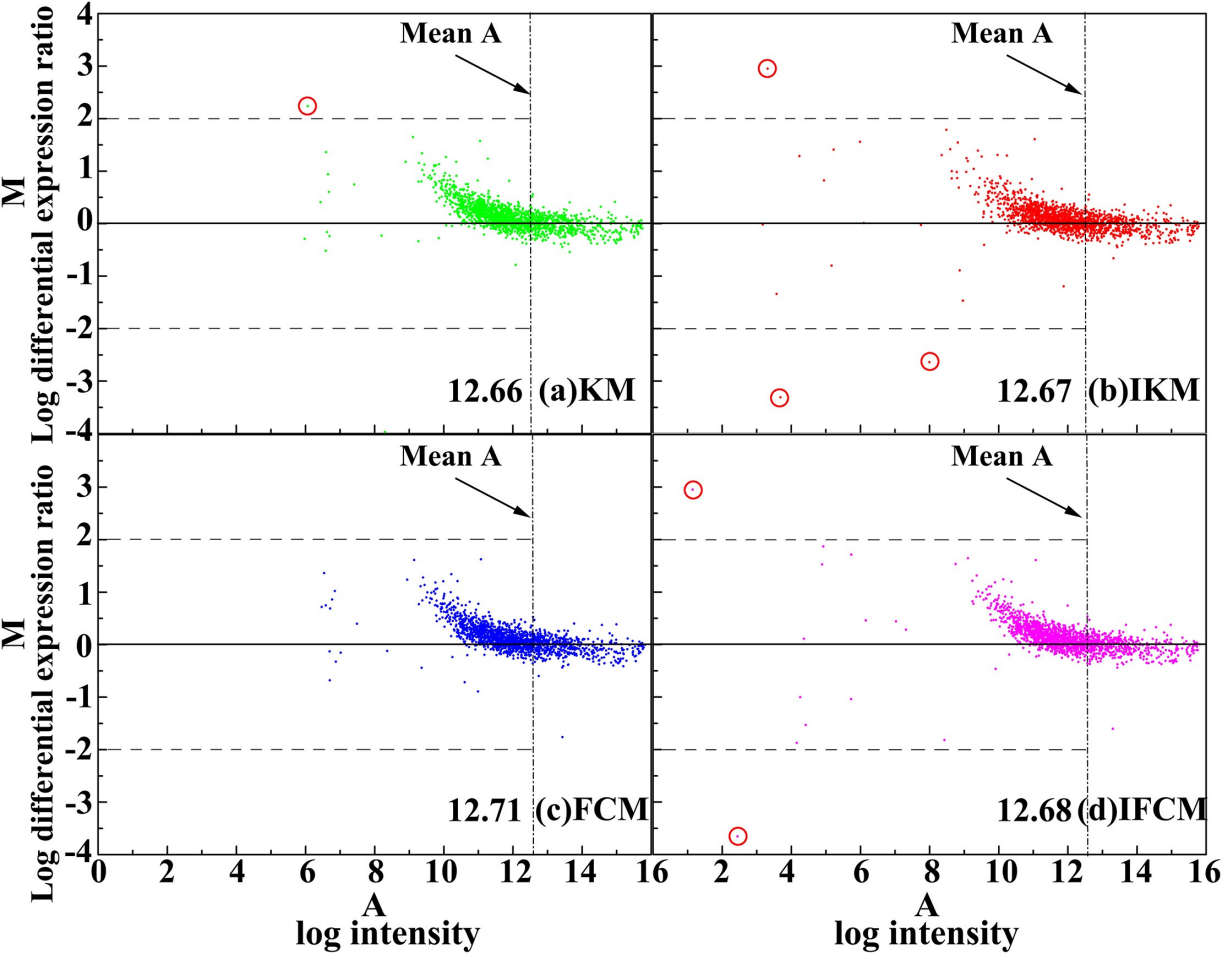
M-A plot of four methods on image drawn from DeRisi dataset.

**Fig 9:**
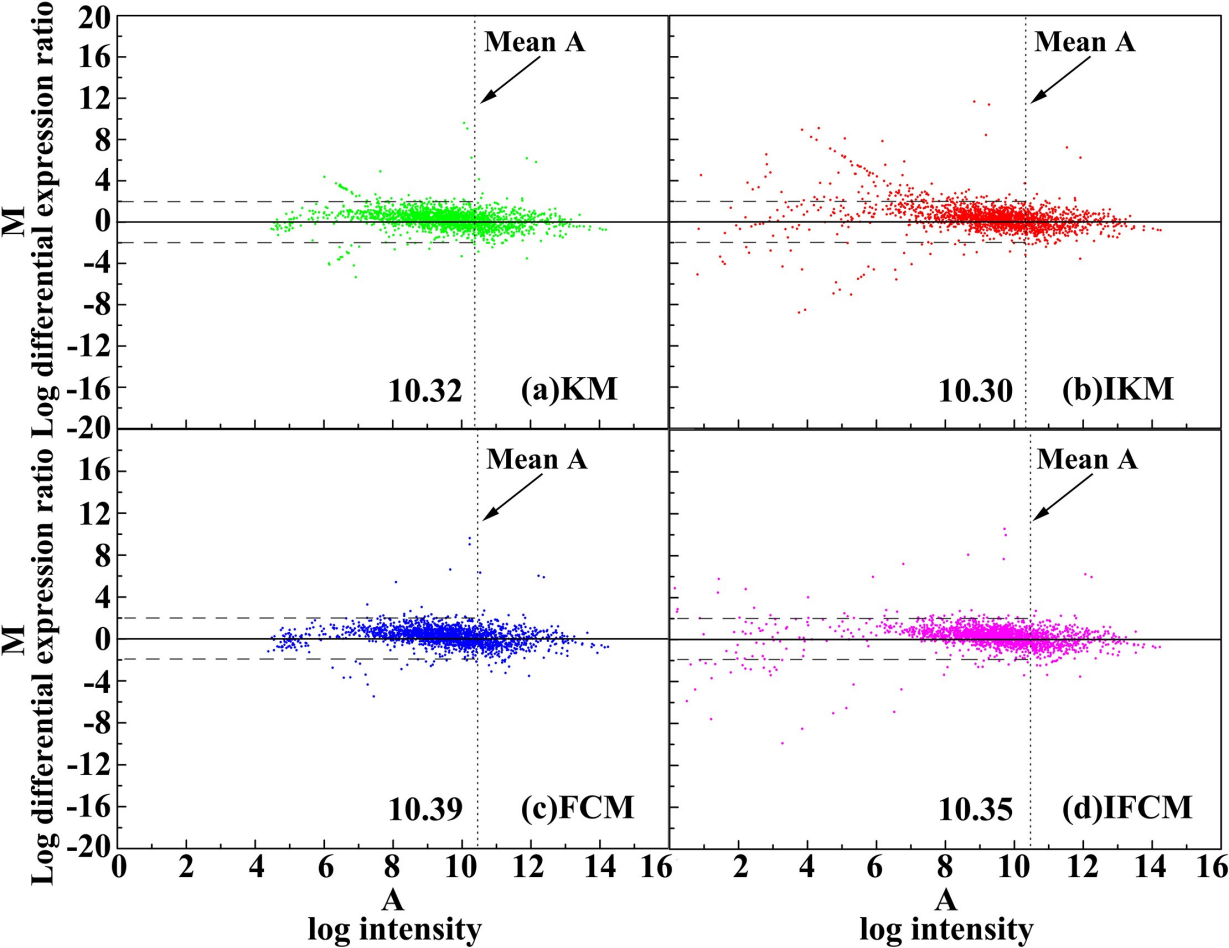
M-A plot of four methods on image drawn from SMD dataset.

Scatter plot method [45] is usually used for describing the correlation between two objects. In general, a straight line in scatter plot indicates that the data points are too closer, *i*.*e*., it is a perfect correlation with their ratio equal to 1. Here, we adopt the scatter plot to compare the background-corrected spot intensities for red channel *I*_*cy*3_ and green channel *I*_*cy*5_. The scatter plot of four methods on DeRisi and SMD datasets results are shown in Figs 6 and 7, respectively. Meanwhile, M-A plot is utilized to diagram the log differential expression ratio M and the log intensity A of each spot, results of four methods on DeRisi and SMD datasets are shown in Figs 8 and 9. Here, to display the results clearly, we convert the original 16-bit gray values into 8-bits on dividing the original data by 256.

In Figs 6 and 7, all data points are exhibited in Red, partial selected data points (to clearly display the correlation between *I*_*cy*3_ and *I*_*cy*5_) are in blue and green, respectively. From Fig 6, it can be seen that the results on the four methods are quite similar and a majority of data points are close to the diagonal line. This phenomenon is in agreement with the analysis on gene expression presented in Fig 5. However, Fig 7 reveals quite different results. First of all, the background corrected intensities of two channels on SMD dataset are much lower than that of DeRisi dataset (Fig 6), that is, most spot intensities are confined around 15 for SMD, yet 60 for DeRisi. In addition, IKM and IFMC algorithms obtain more spots with lower intensities compared to KM and FCM methods (see the circle in Fig 7). In other words, these four methods possess similar performance on spots with higher intensities, while on low contrast spots extracting, only IKM and IFCM methods perform well.

To further prove the effectiveness of our proposed methods, the mean (*MI*_*cy*3_,*MI*_*cy*5_), minimum and maximum background corrected intensities for two channels are calculated, as shown in Table 4. To display the data clearly, we also transferred the data from 16-bit to 8-bit by dividing 256.

**Table 4:** The mean, minimum and maximum background-corrected intensities of four methods for two channels on six datasets.

Method	MI_cy3_	MI_cy5_	Min I_cy3_	Max I_cy3_	Min I_cy5_	Max I_cy5_
	Data set		block name		Spots	
	**GEO**		GSM15898_CH1-1		182	
KM	23.78	24.01	0.54	170.06	0.38	189.80
IKM	23.41	17.74	-0.13	176.32	-0.07	199.57
FCM	25.32	25.55	0.69	178.40	0.50	144.39
IFCM	24.53	18.86	0.71	176.32	-0.02	152.61
	**SMD**		49_ch1-4		1936	
KM	5.20	4.81	0.06	134.34	0.06	94.00
IKM	5.14	4.74	-0.07	140.71	-0.13	99.82
FCM	5.43	5.07	0.05	149.79	0.08	95.62
IFCM	5.29	4.90	-0.01	159.58	-0.02	97.65
	**BCM**		129cy3-1		484	
KM	12.22	13.70	-1.55	132.86	2.75	82.94
IKM	12.32	13.61	2.09	139.62	1.73	81.17
FCM	13.26	14.58	3.88	147.94	3.27	106.93
IFCM	13.03	14.12	3.82	154.32	3.40	130.09
	**DeRisi**		1303_ch1-1		1600	
KM	25.29	25.22	0.22	214.43	0.12	222.41
IKM	25.28	25.53	-0.11	221.59	0.14	224.15
FCM	26.17	26.20	0.32	225.85	0.23	226.55
IFCM	25.72	25.72	-0.006	225.85	0.00	225.05
	**UCSF**		Cy3-4		210	
KM	11.66	10.09	0.03	52.86	0.07	43.95
IKM	11.68	10.10	-0.00	54.01	0.02	44.61
FCM	12.20	10.62	0.03	53.63	0.02	44.54
IFCM	11.91	10.34	0.00	52.26	0.00	45.44
	**SIB**		Def661cy5-1		49	
KM	51.48	89.55	3.06	226.41	9.18	227.90
IKM	51.45	89.23	3.04	226.90	9.16	221.26
FCM	53.21	93.90	3.20	231.62	9.50	236.74
IFCM	53.34	92.46	1.76	233.72	8.61	240.74

One can see from Table 4 that almost all mean background corrected intensities (see MI_cy3_ and MI_cy5_) obtained by IKM and IFCM are smaller than those by KM and FCM on six datasets. The reason is that the proposed IKM and IFCM methods can extract more spots with lower contrast than KM and FCM ones. Meanwhile, the minimum and maximum intensities obtained by IKM and IFCM also exhibit a wider range, implies that our proposed methods perform better on different quality images.

In addition, the M-A plot of the four methods on DeRisi and SMD data set are shown in Figs 8 and 9, respectively. From these two figures, it can be seen that the performance of the four methods are similar for log intensity *A*, all higher than their mean values, but there is remarkably different for the log intensity *A*, smaller than its mean. In other words, for the low log intensity part, IKM and IFCM can obtain more spots with their *M* beyond +2 and -2 than KM and FCM (see circled spots in Fig 8 and spots beyond the dotted line in Fig 9). Actually, spots with higher intensities are easy to extract, yet those spots with lower intensities are difficult to be found. Therefore, IKM and IFCM algorithms may obtain more spots with lower intensities, revealing that these two methods own superior performance. In summary, all these results indicate that the methods of IKM and IFCM perform better than KM and FCM, which proves the effectiveness of our improved strategies.

### Spot segmentation

**Fig 10:**
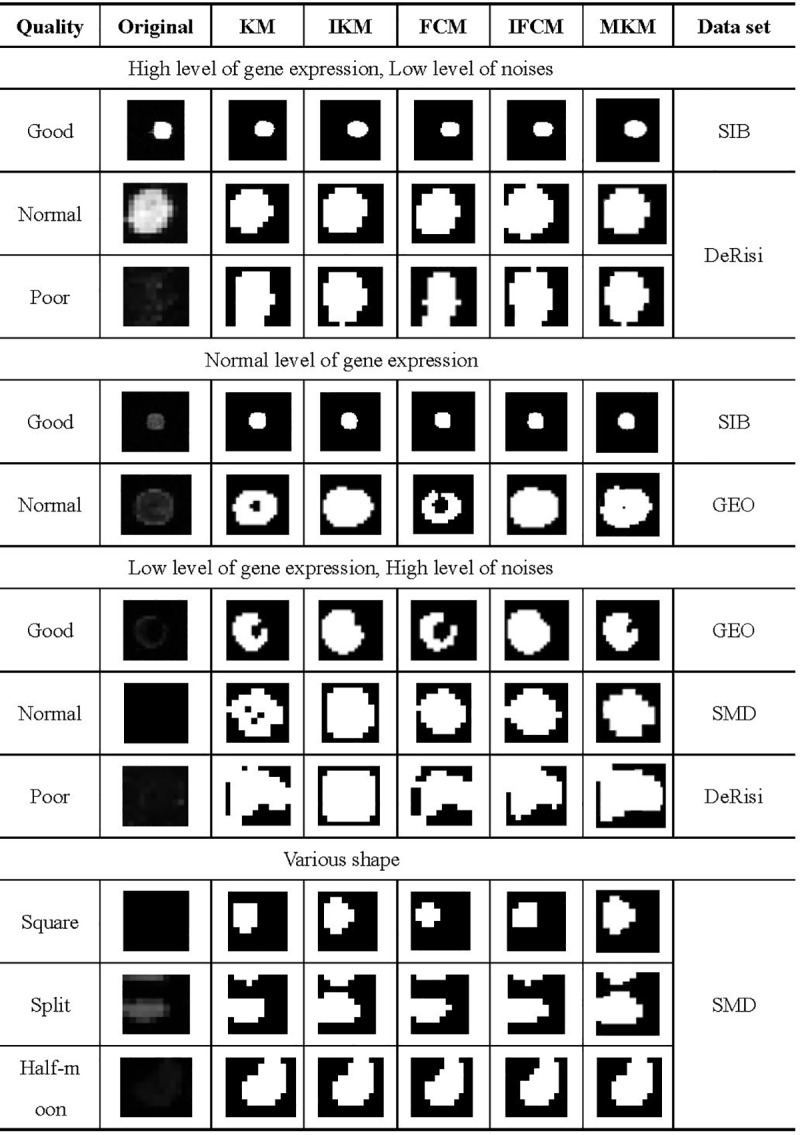
Segmentation results of various shape spots.

As we know, real spots in a microarray image usually contain various shapes instead of circles. Therefore, we choose some spots randomly according to their quality and shape. Examples of these spots and their corresponding segmented binary images are shown in Fig 10. For those good quality spots, no matter what their expression level is, IKM and IFCM algorithms can extract the spots perfectly, yet KM and FCM methods cannot segment the spots completely under the low gene expression level. Obviously, the proposed methods perform better than KM and FCM for both normal and poor quality spots. For MKM, it performs better than KM, but worse than IKM. Similarly, our proposed methods also possess optimal segmented results than KM and FCM on various shape spots.

To further evaluate the effectiveness of our proposed methods, quantitative analysis (pixels segmented as foreground or background, expression level and signal to noise ratio) on above different spots are displayed in Table 5. From Table 5, one can observe that our improved methods (IKM, IFCM) classify more required pixels into foreground (*N*_*fore*_) than KM and FCM algorithms, so as to be closer to the real spot shape. Furthermore, spot intensities for two channels (*I*_*cy*3_,*I*_*cy*5_) obtained by IFCM and IKM are generally lower than those gained from KM and FCM, indicating that IKM and IFCM find more pixels with lower gray values around the spot edge. In other words, KM and FCM algorithms preferentially extract pixels with higher gray values. What’s more, the number of foreground pixels obtained by MKM is similar to IKM and more than KM. Finally, the higher value of signal to noise ratio *q*_*sig*−*noise*_ also indicate the better performance of our proposed methods.

**Table 5:** Quantum analysis of the segmentation results showed in Fig 10.

method	*N*_*fore*_	*N*_*back*_	*I*_*cy*3_	*I*_*cy*5_	*I*_*cy*3_/*I*_*cy*_	*q*_*sig*−*noise*_
**KM**	149	1853	58342	57961	1.01	0.9887
**IKM**	157	1845	56093	58086	0.96	0.9891
**FCM**	140	1862	61630	59832	1.03	0.9888
**IFCM**	144	1858	60606	59294	1.02	0.9893
**MKM**	156	1846	56087	58082	0.96	0.9890
**KM**	151	2404	16483	10325	1.59	0.9710
**IKM**	154	2401	16295	10210	1.59	0.9715
**FCM**	135	2417	17831	11089	1.60	0.9728
**IFCM**	142	2410	17173	11089	1.54	0.9724
**MKM**	152	2403	16338	10289	1.59	0.9713
**KM**	88	294	2680.5	2370.4	1.13	0.8196
**IKM**	135	247	1852.5	2266.8	0.81	0.8533
**FCM**	71	311	3451.7	3020.6	1.14	0.8259
**IFCM**	142	240	1843.7	2569.4	0.71	0.8823
**MKM**	128	254	2178.4	2298.7	0.95	0.8368
**KM**	50	84	46692	40819	1.16	0.9152
**IKM**	54	80	46694	36234	1.28	0.9388
**FCM**	51	83	47387	41684	1.13	0.9252
**IFCM**	66	68	40021	34795	1.15	0.9440
**MKM**	52	82	46693	38174	1.22	0.9217
**KM**	102	280	3036	2338	1.29	0.8517
**IKM**	139	243	2244.4	2161.5	1.03	0.8804
**FCM**	73	309	4117.5	3285.3	1.25	0.8502
**IFCM**	141	241	2212.5	2782.9	0.79	0.9025
**MKM**	128	254	2794.7	2297.6	1.21	0.8638
**KM**	55	47	1788.6	1807.1	0.98	0.8189
**IKM**	56	46	1791.8	1828.4	0.98	0.8234
**FCM**	52	50	1724.8	1828.5	0.94	0.7965
**IFCM**	53	49	1680	1797.6	0.93	0.7919
**MKM**	55	47	1788.6	1807.1	0.98	0.8189
**KM**	60	74	3033.1	3672.3	0.82	0.6920
**IKM**	60	74	2754.4	3198.1	0.86	0.6751
**FCM**	51	83	3117.4	3890.9	0.80	0.6882
**IFCM**	69	65	2753.2	3231.7	0.85	0.6827
**MKM**	60	74	2936.8	3469.5	0.85	0.6931
**KM**	85	49	976.8	1233	0.79	0.5885
**IKM**	87	47	609	1136.3	0.53	0.5532
**FCM**	74	60	943.4	1477.5	0.63	0.5842
**IFCM**	79	55	877.5	1233	0.71	0.5785
**MKM**	86	48	863.4	1194.6	0.72	0.5893
**KM**	19	83	507.3	607.9	0.83	0.7322
**IKM**	23	79	422	92.2	4.5	0.7083
**FCM**	13	89	733.4	367.7	1.99	0.7814
**IFCM**	16	86	605	284.6	2.13	0.7558
**MKM**	21	81	478.2	524.8	0.91	0.7568
**KM**	28	74	13343	14022	0.95	0.9296
**IKM**	39	63	10757	12273	0.87	0.9451
**FCM**	29	73	14178	12935	1.09	0.9529
**IFCM**	32	70	13121	12184	1.07	0.9546
**MKM**	32	70	12866	13242	0.97	0.9362
**KM**	49	83	2781.2	1958.7	1.41	0.8879
**IKM**	49	83	2781.2	1911.6	1.45	0.8879
**FCM**	47	85	2864.5	2871.4	0.99	0.8880
**IFCM**	47	85	2864.5	1800.1	1.59	0.8880
**MKM**	49	83	2781.2	1958.7	1.41	0.8879

### Segmentation results on simulation images

**Fig 11:**
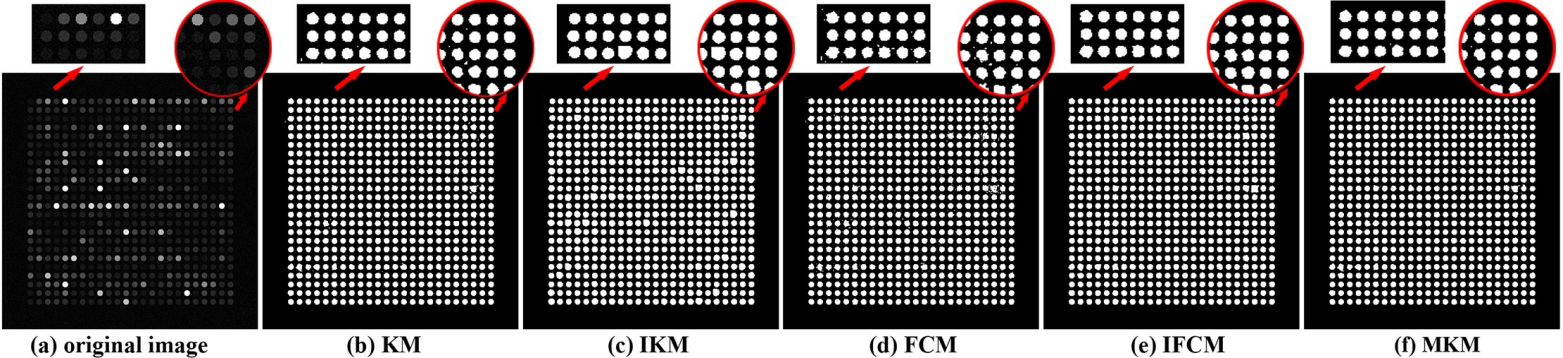
Examples of segmentation results on simulation images.

Because the real images lack of annotation image, we choose simulation images for further comparison. Fig 11 displays the segmented results of four methods. It can be seen that all the spots are extracted from the background for all four methods owing to the fact that simulation image is simple and high quality compared to real image. Meanwhile, as the local magnified parts shown, it is obvious to see that segmentation results of KM and FCM contain lots of noises.

Furthermore, the pixel to pixel accuracy, specificity and sensitivity are computed for each spot and their corresponding means on images in Fig 11, as shown in Table 6. In general, IKM and IFCM own higher values of acc, sp and se than the original KM and FCM, indicating that these two improved algorithms perform better. In addition, the ratio that they cluster the correct pixels into foreground (see *se* in Table 6) are both more than 99%. All these results prove that our proposed IKM and IFCM algorithms outperform than KM, FCM and MKM methods.

**Table 6:** Quantitative evaluation for four methods on simulation image.

method	KM	IKM	FCM	IFCM	MKM
**sp**	0.7839	0.8343	0.7819	0.8388	0.8226
**se**	0.9825	0.9917	0.9775	0.9949	0.9903
**acc**	0.8565	0.8854	0.8540	0.8898	0.8785

### Computational complexity analysis

As we know, the computational complexity of KM is O(n), here n represents the number of spot pixels. Compared to KM, FCM involves computing the Centroid values of each cluster according to its fuzzy membership relation and updating the membership values, so its computational complexity is about several times of KM, As for MKM, there is only one more pixel reassignment operation compared to KM, so their computational complexity is similar. For our improved algorithms, the image contrast enhancement, the multi-features computing, feature selection by PCA and refinement are extra operations compared to KM and FCM, so our methods may be about two or three times over than its original method. Table 7 illustrates the average computational time of above five methods on six data sets. From Table 7 we can see that the same algorithm required various computational times for different data sets due to the distinguished image layout and resolution as displayed in Table 2, in which blocks in UCSF data set take minimum time, yet blocks among BCM data set require maximum time.

**Table 7:** Computational time comparison of five methods on blocks drawn from six data sets (seconds).

method	GEO	SMD	BCM	DeRisi	UCSF	SIB
**KM**	1.829	7.298	16.476	7.111	1.107	2.853
**IKM**	4.004	15.121	36.107	16.258	2.336	5.896
**FCM**	10.159	29.655	99.188	29.064	4.913	15.907
**IFCM**	27.585	79.515	288.847	82.411	13.327	36.804
**MKM**	1.882	7.684	17.099	7.506	1.147	16.472

According to all above analysis, we can draw the following conclusions: for good quality image with high contrast, less noises and sparse spot distribution, the IKM can be used for its segmentation. For poor quality image with low contrast and more noises, IFCM can be utilized to segment the spots. For image contains lots of special shape spots, IKM performs better than IFCM. Finally, IKM and IFCM are both suitable for normal quality image segmentation.

## Conclusions

In conclusion, although current state-of-art clustering-based segmentation methods (KM, FCM) are easy, fast and effective, they are prone to be affected by noises and mostly be conducted based on one feature. Therefore, we proposed the IKM and IFCM algorithms by introducing multi-features such as intensity, spatial, and shape features. Meanwhile, an adaptive adjustment method for segmentation results is introduced and a cluster center initialization strategy is considered. Experiments on six real data sets and one simulation data set verify that our proposed IKM and IFCM methods perform better than the original KM and FCM. In addition, the quantitative analysis on gene expression, background corrected intensities and log-differential expression ratio versus log intensity, further proves the effectiveness of our proposed methods.

## Supporting information

S1 Algorithm (ZIP)

## References

[1] Rajashekhar C.M., Varun S.S. (2015) DNA microarray spot detection using statistical image segmentation. *International Journal of Innovative Research in Computer and Communication Engineering* 3(5):140–147.

[2] Katsigiannis S., Zacharia E., Maroulis D. (2017) MIGS-GPU: microarray image gridding and segmentation on the GPU. *IEEE Journal of Biomedical and Health Informatics* 21(3):867–874. 10.1109/JBHI.2016.2537922 26960232

[3] Shao G.F., Li T.J., Zuo W.D., Wu S.X., Liu T.D. (2015) A combinational clustering based method for cDNA microarray image segmentation. *PLOS ONE* 10(8):1–23. 10.1371/journal.pone.0133025 PMC4524615 26241767

[4] Axon Instruments Inc: GenePix 4000A User's Guide. 1997.

[5] ImaGene, Biodiscovery Inc. [http://www.biodiscovery.com/imagene/].1997.

[6] GSI Lumonics: QuantArray Analysis Software, Operator's Manual. 1999. [http://www.bioprocessonline.com/doc/quantarray-analysis-software-0001]

[7] Eisen M.B. (1997) ScanAlyze. [http://rana.Stanford.EDU/software/]

[8] Heyer L.J., Moskowitz D.Z., Abele J.A., Karnik P., Choi D., Campbell A.M., Oldham E.E., Akin B.K. (2005) Magic tool: Integrated microarray data analysis. *Bioinformatics* 21(9):2114–2115. [http://www.bio.davidson.edu/MAGIC/] 10.1093/bioinformatics/bti247 15647303

[9] Buckley MJ, The Spot user's guide. CSIRO Mathematical and Information Sciences, 2000 [http://www.cmis.csiro.au/IAP/Spot/spotmanual.htm].

[10] Buhler J., Ideker T., Haynor D. (2000) Dapple: improved techniques for finding spots on DNA microarrays UWCSE Tech Report, UWTR Department of Computer Science and Engineering, University of Washington [http://www.cs.wustl.edu/~jbuhler/research/dapple/]

[11] Spotfinder Online Manual, The Insitute for Genomics Research (TIGR), 2004. [http://www.tm4.org/spotfinder.html]

[12] Carlisle A. J., Prabhu V. V., Elkahloun A., Hudson J., Trent J. M., Linehan W. M., et al(2000) Development of a prostate cDNA microarray and statistical gene expression analysis package. *Molecular Carcinogenesis* 28: 12–22. [https://abs.cit.nih.gov/pscan/] 10820484

[13] Jain A.N., Tokuyasu T.A., Snijders A.M., Segraves R., Albertson D.G., Pinkel D. (2002) Fully automatic quantification of microarray image data. Genome Res.12: 325–332. [http://cc.ucsf.edu/jain/public] 10.1101/gr.210902 11827952 PMC155276

[14] Kashyap Ramgopal and Gautam Pratima (2013) Microarray image segmentation using improved GOGAC method. *International Journal of Computer Science and Engineering* 2(4):67–74.

[15] Ni S.H., Wang P., Paun M., Dai W.Z., Paun A. (2009) Spotted cDNA microarray image segmentation using ACWE. *Romanian Journal of Information Science and Technology* 12(2):249–263.

[16] Li Y., P˘aun A., P˘aun M.(2017) Improvements on contours based segmentation for DNA microarray image processing. *Theoretical Computer Science* 701:174–189.

[17] Nguyen H.N., Paveau V., Cauchois C., Kervrann C. (2018) ATMAD: robust image analysis for Automatic Tissue MicroArray De-arraying. *BMC Bioinformatics* 19:148 10.1186/s12859-018-2111-8 29673310 PMC5909283

[18] Ho J., Hwang W.L. (2008) Automatic microarray spot segmentation using a snake-fisher model. *IEEE Transactions on Medical Imaging* 27(6): 847–57. 10.1109/TMI.2008.915697 18541491

[19] Athanasiadis E., Cavouras D., Kostopoulos S., Glotsos D., Kalatzisb I., Nikiforidis G. (2011) A wavelet-based markov random field segmentation model in segmenting microarray experiments. *Computer methods and programs in biomedicine* 104(3):307–315. 10.1016/j.cmpb.2011.03.007 21531035

[20] Zacharia Eleni and Maroulis Dimitris (2011) A spot modeling evolutionary algorithm for segmenting microarray images. *in* *Evolutionary Algorithms*, InTech, 459–479.

[21] Khalilabad N.D., Hassanpour H. (2017) Employing image processing techniques for cancer detection using microarray images. *Computers in Biology and Medicine* 81:139–147. 10.1016/j.compbiomed.2016.12.012 28061369

[22] Deepa J., Thomas T. (2009) Automatic segmentation of DNA microarray images using an improved seeded region growing method. *World Congress on Nature & Biologically Inspired Computing*, *Coimbatore*, INDIA, 1468–1473.

[23] Saadelgawady A., Eltoukhy M.M., Eltawel G., Wahed M.E. (2015) Segmentation of complementary DNA microarray images using marker-controlled watershed technique. *International Journal of Computer Applications* 110(12):30–34.

[24] Thamaraimanalan P., Kumar D.D., Nirmalakumari K. (2014) Efficient gridding and segmentation for microarray images. *International Journal of Computer Science and Mobile Computing* 3(2)):353–360.

[25] Rajkumar P., Vennila I., Nirmalakumari K. (2013) An intelligent segmentation algorithm for microarray image processing. *International Journal on Computer Science and Engineering* 5(6):528–537.

[26] Angulo J. (2008) Polar modeling and segmentation of genomic microarray spots using mathematical morphology. *Image Analtsis & Stereology* 27(2):107–124.

[27] Manjunath S.S., Shreenidhi B.S., Nagaraja J., Pradeep B.S. (2013) Morphological spot detection and analysis for microarray images. *International Journal of Innovative Technology and Exploring Engineering* 2(5):189–93.

[28] Farouk R.M., Badr E.M., SayedElahl M.A. (2014) Recognition of cDNA microarray image using feed forward artificial Neural Network. *International Journal of Artificial Intelligence & Applications* 5(5):21–31.

[29] Giannakeas N., Karvelis P.S., Exarchos T.P., Kalatzi F.G., Fotiadis D.I. (2013) Segmentation of microarray images using pixel classification -Comparison with clustering-based methods. *Computers in Biology and Medicine* 43(6):705–716. 10.1016/j.compbiomed.2013.03.003 23668346

[30] Bozinov D., Rahnenfuhrer J. (2002) Unsupervised technique for robust target separation and analysis of DNA microarray spots through adaptive pixel clustering. *Bioinformatics* 18(5):747–756. 12050071 10.1093/bioinformatics/18.5.747

[31] Belean B. Borda, Ackermann M., Koch J., Balacescu O.I.(2015) Unsupervised image segmentation for microarray spots with irregular contours and inner holes. *BMC Bioinformatics* 16:1–12. 10.1186/s12859-014-0430-y 26698293 PMC4690322

[32] Nagarajan R. (2003) Intensity-based segmentation of microarray images. *IEEE Trans*. *On Medical Imagin*g 22(7): 882–889. 10.1109/TMI.2003.815063 12906242

[33] Rahnenführer J., Bozinov D. (2004) Hybrid clustering for microarray image analysis combining intensity and shape features. *BMC Bioinformatics* 5: 1–11. 10.1186/1471-2105-5-1 15117421 PMC434489

[34] Maguluri L.P., Rajapanthula K., Srinivasu P.N. (2013) A comparative analysis of clustering based segmentation algorithms in microarray images. *International Journal of Emerging Science and Engineering* 1(5):27–32.

[35] Giannakeas N., Fotiadis D.I. (2009) An automated method for gridding and clustering-based segmentation of cDNA microarray images. *Computerized Medical Imaging and Graphics* 33(1):40–49. 10.1016/j.compmedimag.2008.10.003 19046850

[36] Uslan V., Bucak I.Ö. (2010) Microarray image segmentation using clustering methods. *Mathematical & Computational Applications* 15(2): 240–247.

[37] Harikiran J., RamaKrishna D., Phanendra M.L., Lakshmi P.V., Kiran Kumar R. (2012) Fuzzy c-means with bi-dimensional empirical mode decomposition for segmentation of microarray image. *International Journal of Computer Science Issues* 9(3):316–321.

[38] Biju V.G., Mythili P. (2015) Fuzzy clustering algorithms for cDNA microarray image spots segmentation. International Conference on Information and Communication Technologies 46:417–424.

[39] Biju V. G. Myth P. (2017) Possibilistic reformed fuzzy local information clustering technique for noisy microarray image spots segmentation. Current Science 113:1072–1080.

[40] Blekas K., Galatsanos N., Likas A., Lagaris I.E. (2005) Mixture model analysis of DNA microarray images. *IEEE Transactions on Medical Imaging* 24(7):901–909. 16011320 10.1109/tmi.2005.848358

[41] Li Q., Fraley C., Bumgarner R.E., Yeung K.Y., Raftery A.E. (2005) Donuts, scratches and blanks: robust model-based segmentation of microarray images. *Bioinformatics* 21: 2875–2882. 10.1093/bioinformatics/bti447 15845656

[42] Raghavarao S., Madhanmohan M.S., Prasad G.M.V. (2011) Segmentation of microarray image using information bottleneck. *Global Journal of Computer Science and Technology* 11(19):30–33.

[43] Biju V.G. and Mythili P. (2012) A genetic algorithm based fuzzy c mean clustering model for segmenting microarray images. *International Journal of Computer Applications* 52(11):42–48.

[44] National Human Genome Research Institute, http://www.genome.gov, 2009.

[45] Battiato S., Blasi G. D., Farinella G., Gallo G., and Guarnera G. (2007) Adaptive techniques for microarray image analysis with related quality assessment. *Journal of Electronic Imaging* 16(4):043013.

